# Methylphenidate use and poly-substance use among undergraduate students attending a South African university

**DOI:** 10.4102/sajpsychiatry.v22i1.760

**Published:** 2016-03-22

**Authors:** Francois Steyn

**Affiliations:** 1 Department of Social Work and Criminology, University of Pretoria, South Africa

## Abstract

**Background:**

Methylphenidate hydrochloride (MPH) is used in the treatment of attention deficit hyperactivity disorder (ADHD). The non-medical use of MPH by learners and students has been reported by numerous studies from abroad. The practice stems from beliefs about the benefits of MPH in achieving academic success. Little is known about the use of MPH in South African student populations.

**Objectives:**

The study set out to determine (1) the extent and dynamics associated with MPH use and (2) poly-substance use among undergraduate students attending a South African university.

**Methods:**

818 students took part in a written, group-administered survey. Data analysis resulted in descriptive results regarding MPH use and tests of association identified differences in MPH and poly-substance use among respondents.

**Results:**

One in six respondents (17.2%) has used MPH in the past, although only 2.9% have been diagnosed with ADHD. Nearly a third (31.7%) of users obtained MPH products illegally. The majority (69.1%) used MPH only during periods of academic stress. A significant association (*p* < 0.001) was found between MPH use and the frequency of using alcohol, tobacco, cannabis, hard drugs (e.g. cocaine) and prescription medication.

**Conclusion:**

MPH use among students appears similar to experiences abroad, especially in the absence of clinical diagnosis for ADHD. Institutions of higher education should inform parents and students about the health risks associated with the illicit use of MPH. Prescribers and dispensers of MPH products should pay close attention to practices of stockpiling medication and poly-substance use among students who use MPH.

## Introduction

Methylphenidate hydrochloride (MPH) is used in the treatment of attention deficit hyperactivity disorder (ADHD). Prescriptions for and usage of MPH products have increased sharply since the early 1990s with accompanying concerns about potential misuse.^1,2,3^ While peer pressure and experimentation are important factors that contribute to substance use and misuse in young populations, students often experience strain to perform academically. Various strategies are followed to ‘pull all-nighters’ while studying for tests and exams, including the use of MPH to strengthen concentration and the ability to study for prolonged periods of time.^3,4,5,6^ However, evidence is inconclusive whether MPH lives up to the perception of the substance enhancing mental alertness and cognitive performance in persons not clinically diagnosed with ADHD.^2,7^

Self-reported MPH use in student populations ranges from 5% to 34%,^5,8,9,10^ yet only 3% to 10% of MPH users have been diagnosed with ADHD.^3,10,11^ Knowledge about the use of MPH as a study aid appears rife in universities, with up to 44% of students knowing a fellow student who uses MPH for academic reasons.^10^ Characteristics of students who are more likely to use MPH include being white, male, having attended a private school and students who present higher rates of using substances^3,5,6,11^ such as alcohol, tobacco (cigarettes), cannabis, cocaine, ecstasy and amphetamines.^6^

Students use MPH mainly to strengthen concentration, cognition, interest, attention, alertness, energy, focus and memory, to reduce fatigue, to improve academic performance and for recreational purposes.^3,5,6,8,9,10,11^ Very few students use MPH to socialise.^5,11^ Instead, it appears that MPH is used primarily in times of high academic stress (i.e. test weeks and exams).^11^ Procurement of MPH products is reportedly both easy and free from stigma.^3,8^

Students mostly take MPH orally with a small proportion taking MPH intra-nasally.^3,10^ Various side effects of MPH have been reported, including loss of appetite, difficulty sleeping, irritability and a depressed mood.^5^ Nonetheless, students who use MPH show little concern about using or misusing the substance^3^ despite health-threatening consequences if usage is not professionally monitored.^2^

## Aim and methods

The aim of the study was, firstly, to determine the use and dynamics associated with the use of MPH among undergraduate students attending a South African university and, secondly, to determine whether MPH users are more likely to use other substances compared to non-MPH users. Quantitative methods were employed to obtain data from 818 undergraduate students. A group-administered survey design was followed since this strategy ensures anonymity, which was imperative given that students were asked to respond to questions of (illegal) substance use. The survey took place over two days during the undergraduate classes of a discipline that attracts a large number of students annually. Questionnaires and letters of informed consent were distributed in class followed by an explanation of the purpose and procedures of the survey. Students were requested to complete the questionnaire in writing and to not discuss the questions or their answers with fellow students. Students were instructed to fold the completed one-page questionnaires twice so that others could not read their responses during collection.

The questionnaire designed by White et al.^3^ was adapted to reflect local terms (for example ‘first year’ instead of ‘freshman’) and to reduce the 10 open-ended questions to only one. In addition, questions were added about the frequency of students’ use of alcohol, tobacco (cigarettes), cannabis, hard drugs (e.g. cocaine) and prescription medication. MPH use in this survey refers to ‘have ever used’ MPH products with follow-up questions regarding frequency and method of use, sourcing, side effects and openness of use. The questionnaire was piloted with 50 students from other faculties, after which minor alterations were made. Data were captured manually in Microsoft Excel and exported to the Statistical Package for the Social Sciences (version 22).^12^ Inspection of the histograms with normality curves and tests of normality (Kolmogorov-Smirnov *Z*) revealed that the data were not normally distributed. The outcome thus necessitated the use of non-parametric tests to determine significant differences, in particular Pearson’s chi-square and the Mann-Whitney *U* test for which effect sizes (*r*) were calculated.^13^

### 

#### Ethical considerations

The survey adhered to the standard ethical considerations of anonymity, confidentiality and no harm. Respondents were provided with an information sheet which described the purpose and methods of the study, as well as the particulars of organisations that they could contact should they have concerns about their use of substances. Ethical clearance for the survey was obtained from the faculty’s ethics committee and the registrar of the university.

## Results

### Characteristics of the sample and methylphenidate use

The mean age of respondents was 20.38 years (SD = 2.19 years). Female respondents constituted the bulk of the sample and the majority of respondents came from middle-income backgrounds (Table 1). Male and white respondents, as well as those from high-income backgrounds, appear more prone to using MPH. Population group (*r* = –0.25) showed a stronger effect size for MPH use than gender (*r* = –0.07) and economic background (*r* = –0.08).

**TABLE 1:** Background characteristics of respondents and methylphenidate use.

Variable	Total		MPH used		MPH not used		*p*	*r*
		
	*n*	%	*n*	%	*n*	%		
**Gender**								
Male	185	22.7	42	22.7	143	77.3	< 0.001	–0.07
Female	630	77.3	99	15.7	531	84.3	-	-
**Population group**								
Black	244	29.8	9	3.7	235	96.3	< 0.001	–0.25
Mixed-race	48	5.9	7	14.6	41	85.4	-	-
Indian/Asian	28	3.4	1	3.6	27	96.4	-	-
White	499	60.9	124	24.8	375	75.2	-	-
**Economic background**								
Low	72	8.8	6	8.3	66	91.7	0.019	–0.08
Middle	616	75.6	107	17.4	509	82.6	-	-
High	127	15.6	28	22.0	99	78.0	-	-
**Type of school attended**								
Public	567	69.7	90	15.9	447	84.1	0.098	-
Private	247	30.3	51	20.6	196	79.4	-	-

*Source*: Author’s own work.

#### Dynamics of methylphenidate use

Nearly half of all respondents (*n* = 382; 46.8%) know of a fellow student who used MPH. One in six respondents (*n* = 141; 17.2%) has used MPH in the past, although only 2.9% (*n* = 23) of all respondents has been diagnosed with ADHD. Nearly a third (*n* = 33; 31.7%) of MPH users do not have a prescription for the stimulant, with 27.3% (*n* = 30) of users obtaining MPH products from a friend (at an average cost of R35.00 per tablet).

The greater proportion of MPH users started using the substance at university (*n* = 71; 68.3%) and the remainder commenced MPH use at school (*n* = 33; 31.7%). The majority of respondents (*n* = 65; 69.1%) reported using MPH only during tests and exams, with 17.0% (*n* = 16) using MPH daily. A small proportion of respondents use MPH once or twice per week (n = 3; 3.3%), three to four times per week (*n* = 5; 5.4%) and once or twice per month (*n* = 5; 5.4%). The larger part of MPH users indicated that it is very easy (*n* = 47; 41.2%), somewhat easy (*n* = 27; 23.7%) and neither easy nor difficult (*n* = 25; 21.9%) to obtain MPH products. Nine users (7.9%) noted it somewhat difficult and six (5.3%) stated that it is very difficult to obtain MPH products. More than half of users (*n* = 62; 58.5%) indicated that they are open about using MPH, 35.8% (*n* = 38) stated that only those close to them know and six (5.6%) noted that they keep it a secret.

Only one respondent (0.9%) takes MPH intra-nasally with the vast majority taking the stimulant orally (*n* = 106; 99.1%). The reasons for using MPH were to enhance concentration (41.7%), to improve study habits (26.4%), to reduce hyperactivity (10.2%), to achieve higher marks (19.1%) and for socialising (2.1%). MPH users experienced a variety of side effects of which the five most often cited were insomnia (*n* = 13; 17.3%), moodiness (*n* = 11; 14.7%), loss of appetite (*n* = 9; 12.0%), dry mouth (*n* = 6; 8.0%) and anxiety (*n* = 5; 6.7%).

#### Methylphenidate and use of other substances

Although a significant association featured for the co-use of MPH and other substances, tobacco (*r* = 0.24) and cannabis (*r* = 0.21) presented the strongest effect sizes of poly-substance use (Table 2).

**TABLE 2:** Methylphenidate and use of other substances.

Variable	MPH used		MPH not used		*p*	*r*
	
	*n*	%	*n*	%		
Alcohol	120	88.8	526	79.1	0.008	0.09
Tobacco	67	52.3	147	22.8	< 0.001	0.24
Cannabis	57	44.5	127	19.7	< 0.001	0.21
Hard drugs (e.g. cocaine)	7	5.7	5	0.8	< 0.001	0.14
Prescription medication	114	85.1	455	70.0	< 0.001	0.12

*Source*: Author’s own work.

Table 3 cross-tabulates the results of MPH users and non-users with the self-reported frequency of using other substances. MPH users surpassed non-MPH users in the use of other substances in the ‘often’ and ‘sometimes’ categories.

**TABLE 3:** Methylphenidate use and frequency of use of other substances.

Variable	Alcohol		Tobacco		Cannabis		Hard drugs (e.g. cocaine)		Prescription medication	
				
	*n*	%	*n*	%	*n*	%	*n*	%	*n*	%
**Often:**										
Used	43	31.9	33	25.8	16	12.5	1	0.8	35	26.1
Not used	105	15.8	55	8.5	13	2.0	0	0.0	103	15.8
**Sometimes:**																				
Used	49	36.3	14	10.9	11	8.6	3	2.4	36	26.9
Not used	219	32.9	35	5.4	24	3.7	1	0.2	125	19.2
**Seldom:**										
Used	28	20.7	20	15.6	30	23.4	3	2.4	43	32.1
Not used	202	30.4	57	8.8	87	13.5	4	0.6	227	34.9
**Never:**										
Used	15	11.1	61	47.7	71	55.5	116	94.3	20	14.9
Not used	139	20.9	499	77.2	519	80.7	633	98.2	195	30.0
*p*	< 0.001		< 0.001		< 0.001		< 0.001		< 0.001	
*r*	–0.16		–0.25		–0.23		–0.14		–0.15	

*Source*: Author’s own work.

## Discussion

Results from the present survey are similar to findings from abroad regarding the self-reported use of MPH among students (17.2%)^3,10^ and the finding that a small proportion of MPH users has been diagnosed with ADHD (2.7%).^3,10,11^ In the same way, the reasons why students use MPH (mostly to enhance academic performance) and the side effects they experience (notably sleeping disturbances, loss of appetite and moodiness) correspond with existing evidence.^3,5,6,8,9,10,11^ Furthermore, the results show that MPH users are more prone to using other substances,^6^ although MPH poly-use with tobacco and cannabis appears more pronounced. The study further supports findings that white students and those from higher-income backgrounds are associated with higher levels of MPH use,^5,6,11^ possibly due to this profile in South Africa generally having better access to private medical aid and accompanying specialised health care.

Similar to the available evidence, students do not appear to use MPH for socialising purposes but primarily during periods of academic stress.^5,11^ Nearly a third of users did not have a prescription for MPH, thus indicating that they obtain products illegally. These findings raise concerns that students with MPH prescriptions may stockpile their medication over time in order to share it with or to sell it to fellow students. As elsewhere, the results confirm that local MPH users are relatively open about using the substance and that it is relatively easy to obtain MPH products,^3,8^ whether via licit or illicit channels. With nearly half of respondents in the present survey knowing of a fellow student who uses MPH and the majority of users having commenced MPH use at university, it is clear that some form of awareness exists and may be perpetuated on campus about the perceived benefits of MPH as a study aid.

## Recommendations

University management and student support services should create awareness among parents and students about the health risks associated with the misuse of MPH products. Steps should be taken to prevent students with MPH prescriptions from sharing their medication with fellow students.^3^ Medical practitioners and pharmacists should take note of the misuse of MPH among students and carefully follow relevant diagnosis and monitoring protocols in the prescription and dispensing of MPH products, even more so in light of irregular use, sharing and selling of medication and poly-substance use.

## Limitations and future research

The study relied on non-probability sampling procedures and, despite the large number of respondents, generalisation to other settings and populations should be done with caution. Matters of representation are further confounded by not all students attending class, attrition and the bulk of respondents stemming from one specific faculty. Regardless of assurances of anonymity, the study depended on measures of self-report, which is known for under-reporting in matters such as substance use.

Future research, with random and stratified sampling procedures, should focus on MPH use across academic faculties and institutions of higher education. The views of stakeholders, in particular medical practitioners and pharmacists, should be determined regarding the use of MPH among student populations. In addition, a more definite profile of the users of MPH products should be developed in order to identify risk populations and appropriate interventions.

## References

[1] Bogle KE, Smith BH Illicit methylphenidate use: A review of prevalence, availability, pharmacology, and consequences. Curr Drug Abuse Rev 2009;2(2):157–176. PMID: 19630746 10.2174/1874473710902020157

[2] Verster C, Van Niekerk AA Moral perspectives on stimulant use by healthy students. S Afr Med J 2012;102(12):909–911. 10.7196/SAMJ.6090 23498034

[3] White BP, Becker-Blease KA, Grace-Bishop K Stimulant medication use, misuse and abuse in an undergraduate and graduate sample. J Am Coll Health 2006;54(5):261–268. 10.3200/JACH.54.5.261-268 16539218

[4] Babcock Q, Byrne T Student perceptions of methylphenidate abuse at a public liberal arts college. J Am Coll Health 2000;49(3):143–145. 10.1080/07448480009596296 11125642

[5] Rabiner DL, Anastopoulos AD, Costello EJ, Hoyle RH, Swartzwelder HS Motives and perceived consequences of nonmedical ADHD medication use by college students. Are students treating themselves for attention problems? J Atten Disord 2009;13(3):259–270. 10.1177/1087054708320399 18664714

[6] Teter CJ, McCabe SE, Cranford JA, Boyd CJ, Guthrie SK Prevalence and motives for illicit use of prescription stimulants in an undergraduate student sample. J Am Coll Health 2010;53(6):253–262. 10.3200/JACH.53.6.253-262 15900989

[7] Mommaerts J, Beerens G, Van den Block L, et al Influence of methylphenidate treatment assumptions on cognitive function in healthy young adults in a double-blind, placebo-controlled trial. Psychol Res Behav Manag 2013;6:65–74. 10.2147/PRBM.S47526 24039459 PMC3770494

[8] DeSantis AD, Webb EM, Noar SM Illicit use of prescription ADHD medications on a college campus: a multimethodological approach. J Am Coll Health 2008;7(3):315–324. 10.3200/JACH.57.3.315-324 18980888

[9] DuPaul GJ, Weyandt LL, O’Dell SM, Varejao M College students with ADHD. Current status and future directions. J Atten Disord 2009;13(3):234–250. 10.1177/1087054709340650 19620623

[10] Hall KM, Irwin MM, Bowman KA, Frankenberger W, Jewett DC Illicit use of prescribed stimulant medication among college students. J Am Coll Health 2005;53(4):167–174. 10.3200/JACH.53.4.167-174 15663065

[11] Advokat CD, Guidry D, Martino L Licit and illicit use of medications for attention-deficit hyperactivity disorder in undergraduate college students. J Am Coll Health 2008;56(6):601–606. 10.3200/JACH.56.6.601-606 18477513

[12] IBM SPSS Statistics for Windows [computer software] Version 22.0 Armonk, NY: IBM Corp 2014.

[13] Field A Discovering statistics using SPSS. Los Angeles: SAGE; 2009: p. 550.

